# Intelligent spectrophotometric resolution platforms for the challenging spectra of ipratropium and fenoterol in their combination inhaler with ecological friendliness assessment

**DOI:** 10.1038/s41598-024-72431-x

**Published:** 2024-09-28

**Authors:** Salma N. Ali, Samah S. Saad, Ahmed S. Fayed, Hoda M. Marzouk

**Affiliations:** 1 Pharmaceutical Analytical Chemistry Department, College of Pharmaceutical Sciences and Drug Manufacturing, Misr University for Science and Technology, 6th of October City, Giza, Egypt; 2 Pharmaceutical Analytical Chemistry Department, Faculty of Pharmacy, Cairo University, Kasr Al-Aini Street, Cairo, 11562 Egypt

**Keywords:** Chronic obstructive pulmonary disease, Delivered dose uniformity, Fenoterol, Green and white analytical chemistry, Ipratropium, Spectrophotometry, Analytical chemistry, Green chemistry

## Abstract

Asthma and chronic obstructive pulmonary disease (COPD) are the most common diagnoses for adults and children with respiratory tract inflammation. Recently, a novel fixed dose combination consisting of Ipratropium and Fenoterol has been released for the management and control of the symptoms of such disorders. The current research has newly developed and optimized three smart, accurate, simple, cost-effective, and eco-friendly spectrophotometric methods that enabled the simultaneous determination of the drugs under study in their combined inhaler dosage form, without the need for any previous separation steps, using water as a green solvent. The strategy employed was based on calculating one or two factors as a numerical spectrum or constant, which provided the complete removal of any component in the mixture that might overlap and the mathematical filtration of the targeted analyte. The methods developed could be classified into two types of spectrophotometric windows. Window I; involved absorption spectrum in their original zero-order forms (°D), which included recently designed methods named induced concentration subtraction (ICS) and induced dual wavelength (IDW). While window III focused on the ratio spectrum as the induced amplitude modulation (IAM) method. The extremely low absorptivity and lack of distinct absorption maximum in the zero-order absorption spectrum of Ipratropium were two intrinsic challenges that were better overcome by the proposed spectrophotometric methods than by the conventionally used ones. According to ICH guidelines, the proposed methods were validated using unified regression over range 2.0–40.0 µg/mL in the ICS method, while the linearity ranges for the IDW and IAM methods were 5.0–40.0 µg/mL of Ipratropium and 2.0–40.0 µg/mL of Fenoterol. Moreover, the three proposed methods were effectively used to assay the co-formulated marketed inhaler and further expanded to confirm the delivered dose uniformity in compliance with the USP guidelines. Finally, the established methods were evaluated for their greenness and blueness, in comparison to the official and reported analysis methods, using advanced cutting edge software metrics. Furthermore, the suggested techniques adhered well to the white analytical chemistry postulates that were recently published.

## Introduction

Bronchial asthma and chronic obstructive pulmonary disease (COPD) are the most common inflammatory diseases affecting the respiratory tract affecting around 600 million people worldwide^1^. For the symptomatic treatment of airway construction in such patients in both adults and children, bronchodilator treatments administered by inhalation are beneficial^2^. Consequently, the pharmaceutical industry has focused its efforts on managing and controlling the symptoms of both Asthma and COPD disorders by developing new drug mixture, of Ipratropium bromide (IPR) and Fenoterol hydrobromide (FEN)^3^.

**Fig. 1:**
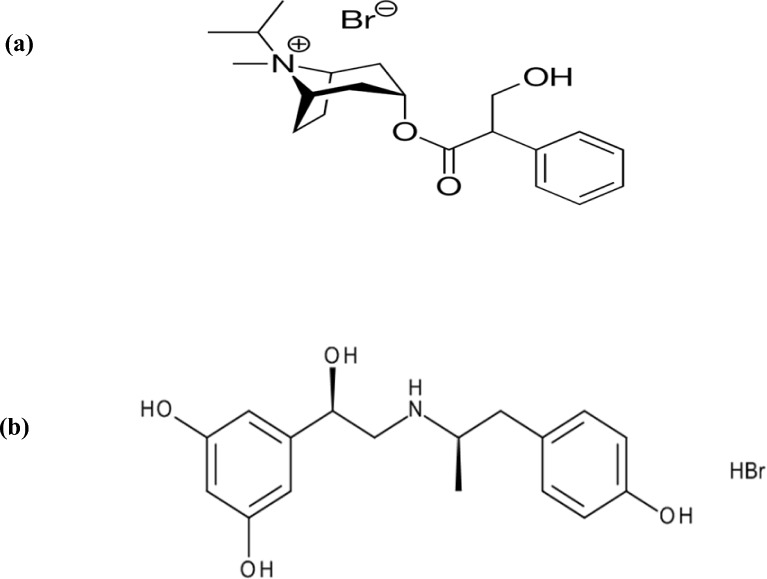
Chemical structures of (**a**): Ipratropium bromide (IPR) and (**b**): Fenoterol hydrobromide (FEN).

IPR is an anticholinergic drug [Fig. 1a] used in the management of symptoms related to bronchospasm. It is a Food and Drug Administration (FDA) approved medication^4^. FEN on the other hand, is a β_2_-adrenergic agonist [Fig. 1b] and categorized as an inhaled bronchodilator asthma medication^5^. Because of their different mechanisms of action, recent study evidence has recommended using IPR in combination with β_2_-agonists for the treatment of acute and severe asthma and COPD, instead of using each drug alone^6^. This combination has been investigated for its ability to improve forced expiratory volume in one second (FEV_1_)^7^. It also effectively provided bronchodilation and decreased the amount of time patients needed to use a metered dose inhaler (MDI)^6,8^. Otherwise, if a lower dose of each medication was taken together, this could produce the same clinical effect, and hence the side effects might be reduced^9^.

The literature displayed only one HPLC method for determination of studied drugs along with other medications in different nebulizer solutions^6^. However, unlike the proposed research, the reported work did not include the assay of the studied co-formulated inhaler (Atrovent® comp HFA) or delivered dose uniformity testing. Additionally, it did not consider the green chromatographic guidelines for analyst safety and environmental preservation.

In the field of instrumental analysis, spectrophotometric methodology is currently regarded as a sustainable and comparable option. It has made significant advance towards establishing quick and efficient resolution methods for drug combinations with overlapping spectra^10–15^. It is superior to other chromatographic methods in being easy to use, quick, and required significantly less time, energy, and solvents consumption^16–18^. In quality control laboratories around the world, spectrophotometry is a widely used technique for drug analysis. Through the use of several spectrophotometric platform windows, it is possible to resolve mixture spectra. These windows are Window I; which deals with the original zero-order absorption spectrum (°D), Window II; which works with derivatized spectrum, Window III; which is focused on the ratio spectrum, and Window IV; which manipulates the ratio derivative spectra^19–21^. Even so, there are drug combinations that frequently exhibit difficulties in their spectra that are unsolvable by the well-known and traditional spectrophotometric methods and requiring the development of more advanced methods.

Therefore, it appears to be more challenging to create a rapid, reasonable, and sustainable spectrophotometric method for the simultaneous analysis of IPR and FEN combination while giving complete adherence to WAC principles. There are three smart spectrophotometric methods established in this work for dealing with the highly complicated and challenging spectrum of the studied mixture, in which traditional manipulation techniques were unable to resolve. In order to manipulate the native zero-order (°D) absorption spectrum (Window I), two methods were used: induced concentration subtraction (ICS) and induced dual wavelength (IDW). The normalized ratio spectrum (Window III) is used in the third technique, termed induced amplitude modulation (IAM). When developing these approaches, extra focus was given to the selection of solvents in order to enhance their economic and sustainable aspects. According to the ICH guidelines, the validity of these techniques, including their accuracy, precision, and specificity, was carefully determined, and they proved to be effective when utilized for assessing the effectiveness of marketed metered dose inhaler. Subsequently, to ensure that every dosage unit has uniform content, the analysis was expanded. This involved randomly choosing different dosage units and performing separate analyses of each one using an appropriate method of analysis^22^. Additionally, it was considered to be an essential prerequisite before beginning a bioequivalence study^23^. The effects of the proposed methods on the environment, human health, and safety were evaluated using valid and cutting-edge tools in addition to the recently developed Blue Applicability Grade Index (BAGI). Ultimately, an excel sheet with the RGB-12 algorithm was used to promote the methods' harmony between analytical, ecological, and practical aspects.

## Experimental

### Instrument and software

Measurements with spectrophotometry were completed using a double beam UV–Vis spectrophotometer (UV-1650; Shimadzu, Tokyo, Japan), equipped with two matching quartz cells with a 1.0 cm path length. UV Probe software version 2.51 was used for data analysis and manipulation. The range of 200.0–400.0 nm was scanned, every 0.1 nm. A scan speed of 2800.0 nm/min was set.

### Chemicals and materials

Pure IPR and FEN were gratefully supplied by Global Napi Pharmaceuticals (GNP) Company (Al-Giza, Egypt). Their potency was checked and found to be 99.40% ± 0.926 and 99.40% ± 1.062 for IPR and FEN, respectively, as per their BP official methods^24^. Atrovent ® comp HFA metered dose inhaler (Batch No. 104604), produced by Boehringer Ingelheim, and is labelled to contain 20.0 µg IPR and 50.0 µg FEN per each metered dose. It was purchased from a local Egyptian pharmacy. Double-distilled water was produced by Aquatron water purification system (A4000D, UK), and served as the main solvent.

### Standard solutions

Stock standard solutions of IPR and FEN were separately prepared with a concentration of 100.0 µg/mL, by accurately weighing 10.0 mg of each pure material and transferring it to a 100-mL volumetric flask followed by full drug dissolution in water, which in the field of analytical chemistry has the highest rating for green solvent. Further dilutions were prepared by taking various volumes (0.5, 1.0, 1.5, 2.0, 3.0, and 4.0 mL of IPR and 0.2, 0.5, 1.0, 1.5, 3.0 and 4.0 mL of FEN) out of the appropriate stock standard solution, transferring each into a 10-mL volumetric flask, and diluting them with water. Stock solutions for IPR and FEN were found to be stable at 4°C in the refrigerator under light protection for up to one week.

### Procedures

Individual aliquots were transferred into two individual sets of 10-mL volumetric flasks from the stock standard solutions of IPR and FEN. To obtain serial dilutions for IPR and FEN within concentration ranges of 5.0–40.0 µg/mL and 2.0–40.0 µg/mL, respectively, volumes were diluted to the flask mark with water. Their °D absorption spectra were measured within a 200.0–400.0 nm wavelength range, using water as blank. Six laboratory mixtures were created by adding different ratio of two drugs along their linearity ranges, and these mixtures were then scanned spectrophotometrically to evaluate the specificity and validity of the method. For additional manipulation events, as detailed under each method for the determination of each drug separately, the recorded absorption spectra were stored. At room temperature, or about (~ 25°C), all experiments were conducted.

#### Induced concentration subtraction (ICS) method

This method involved creating a unified regression equation within a concentration range of 2.0–40.0 µg/mL at a wavelength of 220.0 nm using the recorded °D spectra of pure FEN (extended drug). The two factors of absorptivity (F_1_ & F_2_) are then determined. F_1_ represents the mean of several values calculated by dividing the absorbance values of various concentrations of pure FEN at 220.0 nm (λ_max_) by that at 280.0 nm (λ_ext_). The absorbance value of IPR at 220.0 nm was divided by the absorbance value of FEN at the same concentration to get F_2_. After repeating the procedure for different concentrations, the average value representing F_2_ was calculated. The absorbance value of the drug mixture was recorded at 280.0 nm and multiplying it by F_1_ produced the absorbance associated with FEN in the mixture at 220.0 nm, this was the first step in the analysis process. To estimate FEN concentration, the calculated value was then entered into the unified regression equation. The total drug concentration in the mixture was also estimated using the unified regression equation by directly substituting its absorbance value at 220.0 nm. To determine the IPR concentration in the mixture, the obtained FEN concentration will be divided by F_2_ after subtracting from the mixture's total concentration.

#### Induced dual wavelength (IDW) method

For each drug, a unique manipulation protocol was developed. Using the °D spectra of pure IPR, representing the interfering drug, an equality factor (E.F_IPR_) was first calculated in relation to FEN determination. This can be calculated by dividing the absorbance value of pure IPR at 220.0 nm by that at 210.0 nm for a different number of pure IPR concentrations, then find the average value. A linear regression equation was created to estimate the concentration of FEN over a range of 2.0–40.0 µg/mL. This was achieved via plotting the absorbance difference between FEN absorbances at 220.0 nm and 210.0 nm after the multiplication by E.F_IPR_ [ΔA = A_220_ $-$ (A_210_ $\times$ E.F_IPR_)] against the related FEN concentration. In this manner, the concentration of FEN in a mixture could be readily determined by measuring the °D absorbance at 220.0 and 210.0 nm of the mixture, computing ΔA, and then making the needed substitutions in the corresponding regression equation. The method for determining IPR remained the same, but a linear regression equation covering the concentration range of 5.0–40.0 µg/mL of IPR was created. Using the pure FEN °D spectra at 210.0 and 220.0 nm, the equality factor (E.F_FEN_) was computed.

#### Induced amplitude modulation (IAM) method

The starting point of this procedure was to use UV-Probe software to divide the entire °D spectra by its concentration value (20.0 µg/mL) to obtain "normalized spectra" for each FEN and IPR. The second step established by dividing the obtained FEN normalized spectrum by the IPR normalized spectrum, a new spectrum known as the "absorbance ratio spectrum" was created. The third step involved using the corresponding normalized spectrum as a divisor to draw ratio spectra of °D spectra of pure FEN or IPR. There were two developed linear regression equations: the first [Eq. (1)]; correlated the amplitude values of the FEN ratio spectra at 210.0 nm with the associated concentrations in the range of 2.0–40.0 g/mL, whereas the Second Equation [Eq. (2)] explored the relationship between the amplitude values of the IPR ratio spectra at 210.0 nm with the corresponding concentrations in the range of (5.0–40.0 g/mL). Using the "normalized spectrum" of FEN, the fourth step involved adjusting the ratio spectra of pure IPR once again. The amplitude difference between wavelengths 210.0 and 220.0 nm (ΔP = P_1_ − P_2_) was plotted against their proposed amplitude at 210.0 nm (P_1_), allowing for the establishment of a linear regression equation [Eq. (3)]. The ratio spectrum was adjusted to quantify the drug mixture using the FEN's "normalized spectrum" as a divisor. Equation (3) was utilized to estimate the P_1_ value by substituting the amplitude difference (ΔP), which was obtained by calculating the wavelength difference between 210.0 and 220.0 nm. Subtraction of the recorded amplitude value of the mixture at 210.0 from the estimated P_1_ value yields a constant value. This value served two purposes: first, it was used to estimate the concentration of FEN in a mixture by directly substituting it into Eq. (1); second, the value was subtracted using UV-Probe software from the mixture's entire ratio spectra. To obtain the IPR concentration in the mixture, the amplitude value at 210.0 nm was substituted in Eq. (2) after multiplying the obtained spectrum by the "absorbance ratio spectrum" that was stored.

### Application to marketed co-formulated metered dose inhaler

Two milliliters of the metered dose inhaler solution (Atrovent® comp HFA), representing 800 µg IPR and 2000 µg FEN, were accurately placed into a 10-mL volumetric flask. Mixed and diluted to the flask mark with water to reach the final concentration of 80.0 µg/mL IPR and 200.0 µg/mL FEN. For each of the estimated analytes, further dilutions with water were applied to achieve concentrations through the linearity ranges. The methods recommended were then applied to determine each drug's concentration. Additionally, the standard addition technique was employed. This required adding exactly appropriate amounts of pure IPR and FEN to the dosage form solution and then diluting the mixtures with water. The steps were performed exactly as explained for each approach.

### Testing the delivered dose uniformity

The methods provided were also used to assess the delivered dose uniformity of the marketed inhaler in accordance with international guidelines^25,26^. The analysis was carried out in the same manner as previously described to determine IPR and FEN in the Atrovent® comp HFA inhaler with the exception that only two actuations (dosage unit) were placed into a 5-mL volumetric flask and diluted to the flask mark with water. Following the direct recording of absorption spectra, the studied drug concentrations were calculated using the aforementioned techniques. After that, the procedures were repeated ten times, each time with a new delivered dosage unit.

## Results and discussion

**Fig. 2:**
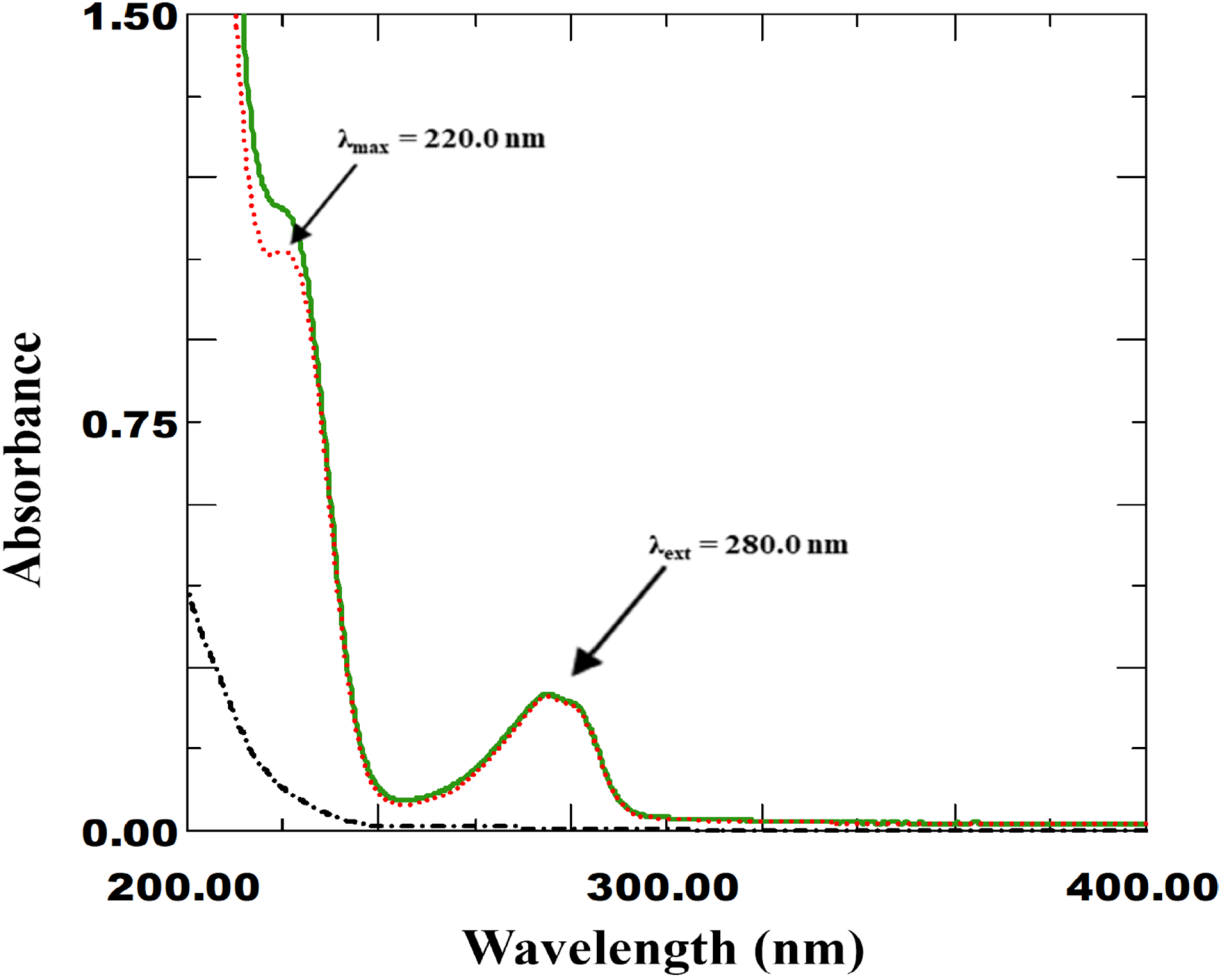
Overlain zero-order absorption spectra of 8.0 µg/mL IPR (-⋅-⋅-) and 20.0 µg/mL FEN (**….**) separately in distilled water as well as their synthetic mixture 8.0 µg/mL IPR and 20.0 µg/mL FEN (**−**), showing the maximum (λ_max_) and extended (λ_ext_) wavelengths for FEN.

In the field of drug analysis and quality control, spectrophotometry has attracted a lot of attention due to its high reproducibility, ease of use and low solvent and energy consumption per sample analysis^27^ in the direction of a clean and green environment. Spectrophotometric technique has surpassed common chromatographic and electrochemical techniques ones for monitoring the potency and content of pharmaceutical formulations available on the market^12^. However, a newly reported mathematical filtration approach is required because the extreme overlap of spectra and many challenges in drug mixture make it difficult to directly quantify each of its individual components^28,29^. Through the development of three distinct and ingenious resolution approaches, Spectrophotometric analysis was used for the first time to determine the newly co-formulated mixture of IPR and FEN. When the °D absorption spectra of IPR and FEN were recorded, both individually and together, FEN expressed peak maxima at 220.0 nm (Fig. 2). Figure 2 illustrated a spectral extension of FEN over the IPR spectrum, with a peak maximum (λ_ext_) at 280.0 nm in a wavelength range of 279.0–400.0 nm. The figure in this mixture also evoked several spectral challenges. The IPR spectrum was blamed for the majority of them because of its lack of distinguishing peaks, extremely low absorptivity and absence of measurable absorbance except at shorter wavelengths less than 250.0 nm, in which the majority of organic solvent interferes additionally to having the least amount of substance in the dosage form. Another limitation was the low absorptivity of FEN at λ_ext_ 280.0 nm, its extended region, where quantification exhibited extremely low precision and sensitivity. Due to all these limitations, it was impossible to directly quantify it at the extended region, which made the use of additional artistic approaches to manipulation necessary. To overcome all these challenges, initial efforts were focused on applying traditional spectrophotometric techniques. The precise drug quantification by different derivatization techniques was, however, limited by not having any zero-crossing, zero-contribution, or both at various derivatization orders, from first to forth. Many techniques, including absorbance subtraction, absorptivity centering, amplitude modulation, and advanced amplitude modulation, were excluded because of the lack of iso-absorptive points^30–37^. Moreover, the lack of two wavelengths with zero absorbance difference restricted the simple dual wavelength approach^32,36,44,45^. Consequently, as the following lines will show, this work was devoted to implementing a newly established approach through the application of one or more numerical factors and spectrum to create mathematical filtration.

### Solvent optimization and selection

The primary challenge for any spectrophotometric method is selecting an appropriate solvent that displays two essential attributes: a lower UV-cut off value and be able to dissolve all target analytes. Every solvent has a wavelength below which it absorbs light and starts to interfere with the target analyte. This is known as the absorbance cut off. This problem is particularly noticeable when spectrophotometrically analyzing our mixture because IPR has low UV absorptivity and only UV absorbance at shorter wavelengths (< 250.0 nm), as shown in Fig. 2. To enable IPR determination at shorter wavelengths without solvent interference, a solvent with an extremely low UV cut-off, such as methanol, acetonitrile and water, was chosen^38^. Water is considered to be the least expensive and most environmentally friendly organic solvent when taking the GAC principles with regard^39,40^. Water was chosen as the solvent for this work because it is cheap, readily available, sustainable, and safe in addition to having a strong dissolution power for the drugs under study.

### ICS method (Window I)

One of the newest methods for solving overlapped mixture spectra at 220.0 nm with an individual regression equation is ICS^41,42^. The ICS method handles °D absorption spectrum in several steps on the same spectra without the need for a divisor or extra derivatization steps by using an exact and creative mathematical protocol. As a result, ICS falls into the categories of progressive resolution technique and Window I spectrophotometric platform^19^. In this approach, two wavelengths were chosen: 280.0 nm, which represents the λ_ext_ of FEN, and 220.0 nm, which represents the λ_max_ of FEN with a specific absorbance of IPR (Fig. 2). Using FEN absorbance at λ_max_ (220.0 nm) over a concentration range of 2.0–40.0 µg/mL, a unified regression equation was created. Even though FEN was more extended than IPR, it exhibits extremely low absorbance in the extended region over 280.0 nm, making it impossible to analyze FEN at the extension region because it is not robust or sensitive enough. To exploit this expanded region, this proposed method calculates two factors; The first absorptivity factor (F_1_) for pure FEN representing the relation between the absorbance at λ_max_ of FEN (220.0 nm) and that at extended region with no contribution of IPR (280.0 nm), and it was found to be 4.70. The second factor (F_2_), on the other hand, was estimated to be 0.20 and was intended to correlate the absorbance value of pure IPR and FEN with the same concentration at the λ_max_ (220.0 nm). Consequently, multiplying F_1_ by the mixture absorbance at 280.0 nm resulted in the estimation of the absorbance contribution of FEN in the mixture. The FEN concentration was then calculated by substituting the obtained value into the unified regression equation. However, the total mixture concentration can be estimated by substituting the mixture absorbance at 220.0 nm. The obtained FEN concentration in the mixture was simply subtracted from the total concentration of the mixture to obtain the IPR concentration, but from the viewpoint of FEN absorptivity. Consequently, the final step involved dividing it by 0.20 (F_2_) to restore the IPR absorptivity constant and correct the concentration.

### IDW method (Window I)

**Fig. 3:**
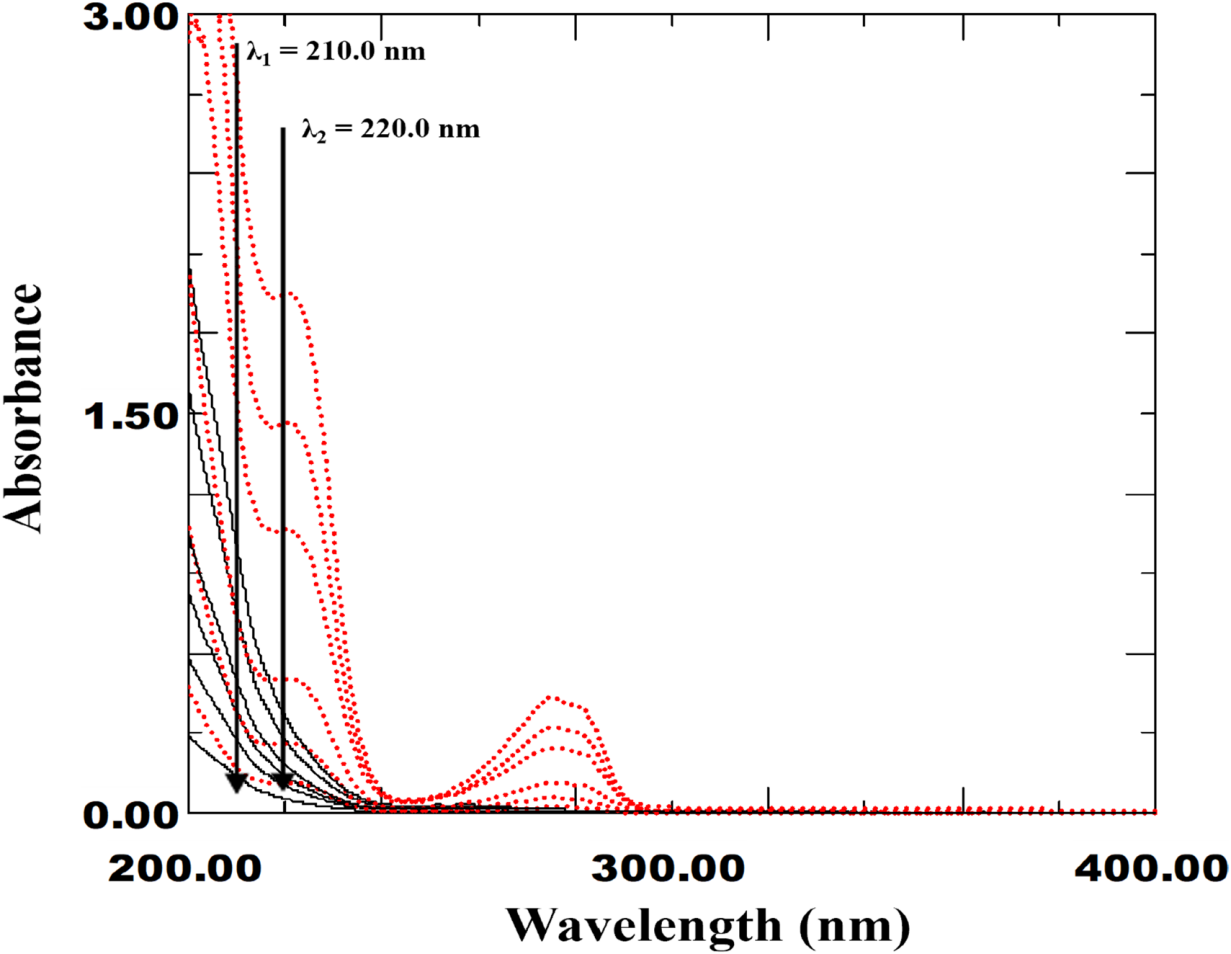
Zero-order absorption spectra of various concentrations of IPR (**−**) and FEN (**….**) showing absorbance difference at 210.0 nm and 220.0 nm [IDW method].

The core concept of this approach is the elimination of the absorbance of the interfering substance between two chosen wavelengths. This method is used in cases where the absorbance difference (DA) of the interfering substance is not equal to zero, in contrast to the traditional dual wavelength method (DWM)^32,36,45^. For determining the component of interest (FEN), the interfering component (IPR) should be cancelled at two chosen wavelengths. The traditional dual wavelength approach could not be used for FEN by screening the °D spectra of both components because the IPR spectrum does not contain two points with the same absorbance. The interfering component (IPR) could therefore have zero absorbance difference at two selected wavelengths (λ_1_ and λ_2_) as a result of the development of IDW^43–45^. The "Equality factor, E.F." was calculated by dividing the absorbance value of the interfering component (IPR) at λ_1_ by its absorbance at λ_2_, which was done in order to cancel the absorbance value of the IPR at the two selected wavelengths, even though the absorbance of the component of interest (FEN) will differ. It is important to remember that induced mathematical filtration commonly referred to as the (progressive resolution technique) is carried out in steps on the same spectrum using °D absorption spectra (Window I spectrophotometric platform)^19^. This study chose λ_1_ of 220.0 nm due to its high absorbance for the drug of interest (FEN) and distance from the solvent UV-cut off. A study of optimization was carried out to determine the optimal value of λ_2_, varying it between 210.0 and 230.0 nm. The equality factor for various concentrations of the interfering drug (IPR) was then calculated. The wavelength that had the lowest RSD% value was 210.0. To determine the optimal value of λ_2_, another optimization study was conducted by plotting ΔA (ΔA = A_220_ – E.F × A_λ2_) against the associated target drug concentration and comparing the resulting slope, which indicates sensitivity of the desired drug. It was discovered that a wavelength of 210.0 nm provided adequate sensitivity. Therefore, 210.0 nm and 220.0 nm were the two chosen wavelengths for the two drugs (Fig. 3). In order to determine the FEN, the linear regression function generated and E.F_IPR_ value of 0.382 was calculated for concentrations of 2.0–40.0 µg/mL of FEN. On the other hand, the IPR was determined within the range, 5.0–40.0 µg/mL, and an E.F_FEN_ value of 1.37 was calculated. The analysis of the drug mixture was then performed simply by measuring its absorbance at 210.0 and 220.0 nm. After estimating ΔA (A_220_ – E.F_IPR_ × A_210_) and substituting it into the FEN regression equation, the concentration of FEN in the mixture was determined. In contrast, the IPR concentration was calculated by changing the corresponding regression equation to ΔA (A_220_ – E.F_FEN_ × A_210_).

### IAM method (Window III)

**Fig. 4:**
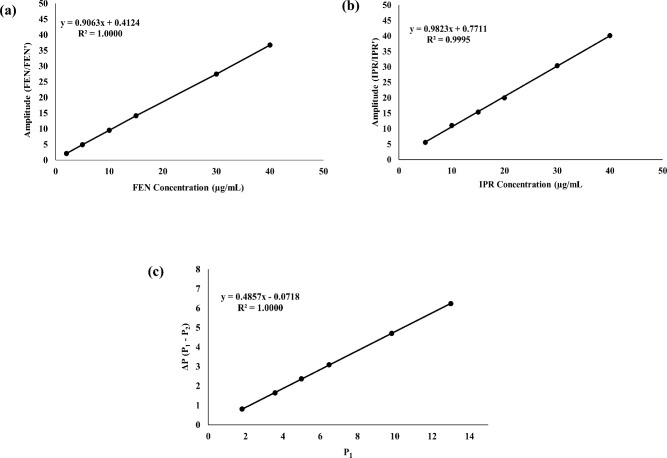
Linear regression equations for amplitude values at 210.0 nm of (**a**): FEN ratio spectra using normalized FEN spectrum as a divisor versus FEN concentrations and (**b**): IPR ratio spectra using normalized IPR spectrum as a divisor versus IPR concentrations. (**c**) Correlation formula between amplitude differences at 210.0 & 220.0 nm versus amplitude values at 210.0 nm of IPR ratio spectra using normalized FEN spectrum as a divisor [IAM method].

**Fig. 5:**
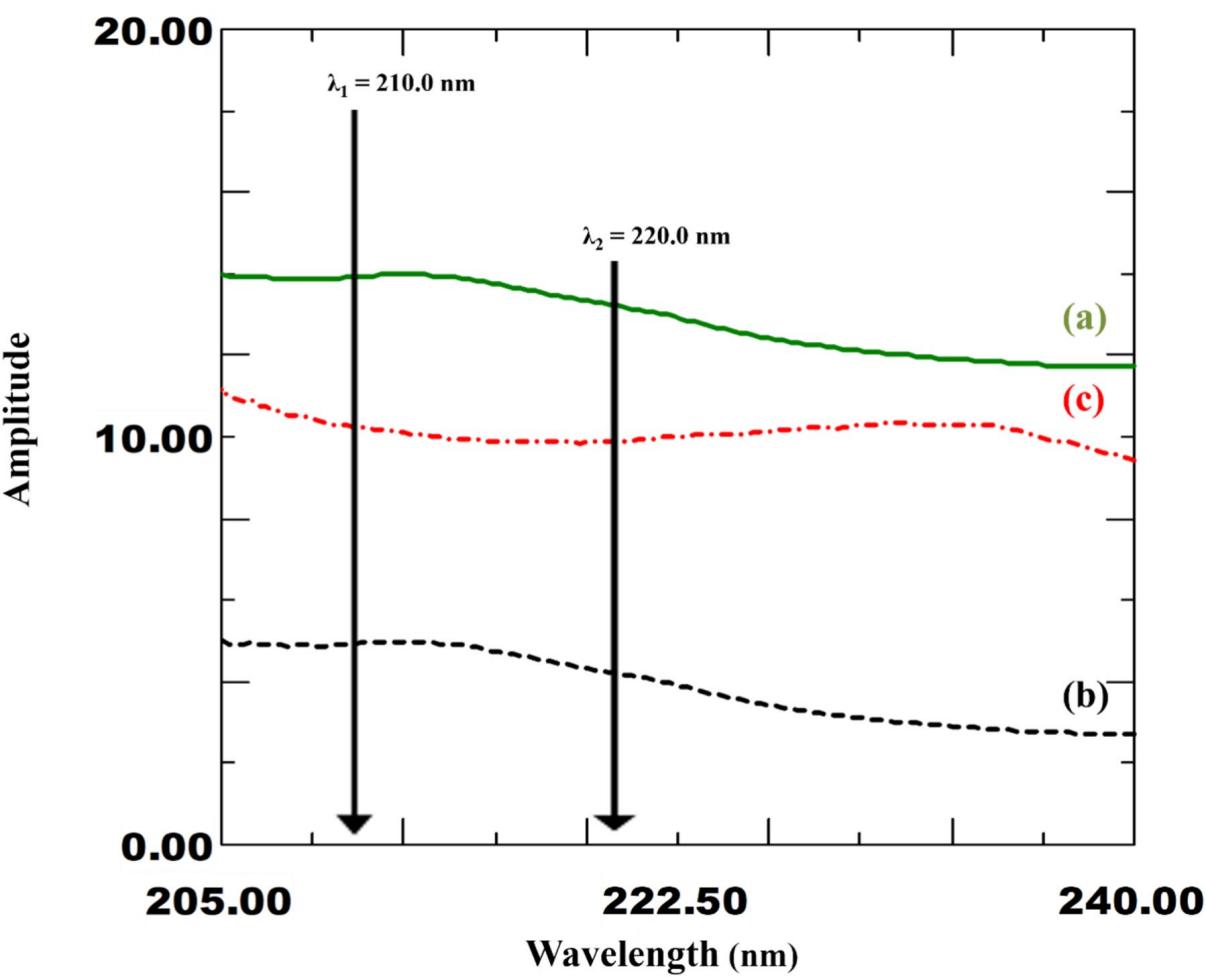
Steps of IAM method: (**a**) Ratio spectrum of synthetic mixture containing 10.0 μg/mL IPR & 5.0 μg/mL FEN utilizing normalized FEN spectrum as a divisor. (**b**) Resolved ratio spectrum of 10.0 μg/mL IPR obtained after subtracting FEN/FEN’ constant. (**c**) Modulated amplitude spectrum equivalent to concentration of IPR (10.0 μg/mL) in the mixture, after multiplying by the absorptivity ratio spectra of FEN’/IPR’.

The IAM method could be considered as a more advanced and broadly applicable version of the "amplitude modulation" method^34,35^. The theory behind the IAM method is rooted in the original amplitude modulation method, which modulated amplitude to the corresponding concentration by using normalized spectra as divisors^41,43^. However, because it requires both a spectral extension of one component over the other and the existence of an isosbestic point with a significant amplitude, the amplitude modulation method is limited in its applications. Although °D of FEN shows extension over IPR, the original amplitude modulation approach, which required one of the drugs to have an extended region, failed to achieve desirable results. All these limitations were eliminated in IAM, as an enhanced version, improving its capacity to resolve complex mixtures devoid of any spectral features. The only requirement for choosing the wavelengths is that each mixture component has enough amplitude at both wavelengths^46^. Other than that, no special criteria are needed. Two wavelengths were used in our work: 210.0 nm (λ_1_) and 220.0 nm (λ_2_). The corresponding normalized spectrum (FEN' or IPR', respectively) was used to draw the ratio spectra of each concentration of FEN or IPR separately. To create two regression equations, amplitude values $(\frac{FEN}{{FEN^{\prime}}}{\mkern 1mu} or{\mkern 1mu} {\mkern 1mu} \frac{IPR}{{IPR^{\prime}}})$ at 210.0 nm were plotted against the corresponding concentrations of FEN in the range of 2.0–40.0 µg/mL (Eq. (1), Fig. 4a) and IPR in the range of 5.0–40.0 µg/mL (Eq. (2), Fig. 4b). Additionally, using the normalized spectrum of FEN as a divisor (FEN'), ratio spectra of various IPR concentrations were drawn. Equation (3), Fig. 4c, was created by utilizing the above ratio spectra of IPR to create a correlation between the amplitude difference at 210.0 & 220.0 nm $\left( {\Delta {\text{P}} = \frac{{IPR_{1} }}{{FEN^{\prime}}} - \frac{{IPR_{2} }}{{FEN^{\prime}}}} \right)$ and the amplitude value at 210.0 nm $\left( {P_{1} = \frac{{IPR_{1} }}{{FEN^{\prime}}}} \right)$. Using the normalized spectrum of FEN (FEN') as a divisor, the amplitude values of ratio spectra of the drug mixture were recorded at two wavelengths (210.0 & 220.0 nm), as shown in Fig. 5a. The difference between the two values has been substituted in Eq. 3 to obtain the postulated $P_{1} \,\,\left( {\frac{{IPR_{1} }}{FEN^{\prime}}} \right)$at 210.0 nm. This was then subtracted from the mixture's recorded amplitude value at 210.0 nm $\left( {\left[ {\frac{{FEN^{\prime}}}{{FEN^{\prime}}}{\mkern 1mu} + \frac{{IPR_{1} }}{{FEN^{\prime}}}} \right]{\mkern 1mu} - \frac{{IPR_{1} }}{{FEn^{\prime}}}} \right)$, yielding a constant value $\left( {\frac{{FEN_{1} }}{{FEN'}}} \right)$. After that developed, the constant value that was obtained equaled the concentration of FEN in the mixture. This constant value was substituted in Eq. (1) to determine the actual FEN concentration in the mixture. However, by subtracting this constant $\left( {\frac{{FEN_{1} }}{{FEN'}}} \right)$ from the entire mixture ratio spectrum, the IPR ratio spectrum $\left( {\frac{{IPR}}{{FEN'}}} \right)$ can be gained (Fig. 5b). Then, this spectrum was multiplied by the "Absorbance ratio spectrum" which was calculated by dividing the entire FEN normalized spectrum by the IPR normalized spectrum $\left( {\frac{{FEN'}}{{IPR'}}} \right)$. Figure 5c shows the spectrum ($\left( {\frac{{IPR}}{{IPR'}}} \right)$), with amplitude values modulated to the IPR concentration in the mixture. The exact IPR concentration in the mixture was calculated by substituting the amplitude value at 210.0 nm using Eq. (2).

Complete theory and explanation of the proposed methods, including step-by-step mathematical equations, are displayed in the *Supplementary File*.

### Developed methods’ validation

According to ICH guidelines^47,48^, various performance parameters were analyzed to confirm the validity of the proposed methods. Based on the construction of calibration curves over three successive days, a working linearity range was determined for the proposed approaches. The ICS method displayed good linearity within the range of 2.0–40.0 µg/mL, whereas the IDW and IAM methods showed linearity within the ranges of 5.0–40.0 µg/mL and 2.0–40.0 µg/mL for IPR and FEN, respectively. Acceptable linearity was indicated by small intercept values and correlation coefficients for six concentration levels ≥ 0.999. Table 1 provided illustrations for each parameter in the regression equations. To ensure the accuracy of the methods, five concentration levels of each drug (6.0, 12.0, 18.0, 25.0, and 35.0 µg/mL) in triplicates within the known linearity range of the drugs under study were used. In addition, three different levels of 5.0, 10.0, and 30.0 µg/mL, in triplicates, were used to assess repeatability and intermediate precision on the same day and three days consequently, respectively. The satisfactory recovery percentage within the acceptance criteria for assay of an active ingredient (98–102%) and the small relative standard deviations (%RSD) less than two, obtained in Table 1 demonstrate the good accuracy and precision of the suggested methods. To assess the impact of changing one of the methods' parameters on their analytical performance, a robustness study was also carried out. Using distilled water from various sources, triplicate analyses of three different drug concentrations were performed with acceptable (%RSD) value < 2% as summarized in Table 1. Furthermore, limit of detection (LOD) and quantification (LOQ) were calculated for the cited drugs^47^. The values obtained demonstrate the good sensitivity of the proposed methods, Table 1. Lastly, by creating numerous laboratory-prepared mixtures with varied drug concentrations, the ability to quantify the studied drugs in binary mixtures, simultaneously, with different composition ratios was evaluated. In order to shed light on the specificity of the presented methods, Table 2 displays the analysis results of acceptable percent recoveries and (%RSD) values with the absence of interference.

**Table 1:** Assay parameters and method validation for the determination of Ipratropium and Fenoterol pure samples by the proposed spectrophotometric methods.

Parameter	ICS method	IDW method		IAM method	
	IPR or FEN	IPR	FEN	IPR	FEN
Wavelength (nm)	220.0	210.0 & 220.0	210.0 & 220.0	210.0	210.0
Linearity Range (µg/mL)	2.0–40.0	5.0–40.0	2.0–40.0	5.0–40.0	2.0–40.0
-Slope	0.0487	0.0126	0.0238	0.9823	0.9063
-SE of the slope	0.0001	0.0001	0.0001	0.0110	0.0030
-Intercept	0.0185	-0.0053	0.0022	0.7711	0.4124
-SE of the intercept	0.0027	0.0015	0.0032	0.2561	0.0657
-Correlation coefficient (r)	1.0000	0.9999	0.9999	0.9997	1.0000
Accuracy ^a^ (Mean ± RSD%)	100.67 ± 1.289	100.27 ± 1.175	98.91 ± 0.825	100.04 ± 0.609	100.69 ± 0.989
Robustness (RSD%)	1.070	1.045	0.972	1.106	0.892
Precision ^b^ (RSD%)					
-Repeatability	0.797	0.644	0.973	0.432	0.358
-Intermediate precision	1.019	1.014	1.208	0.955	0.787
LOD (µg/mL)	0.225	0.408	0.555	0.880	0.300
LOQ (µg/mL)	0.683	1.236	1.682	2.666	0.909

^a^Average of five determinations.

^b^Average of three determinations.

**Table 2:** Determination of Ipratropium and Fenoterol in laboratory prepared mixtures by the proposed spectrophotometric methods.

Mixture No	Claimed concentration (µg/mL)			Recovery %							
				ICS method			IDW method			IAM method	
	IPR	FEN		IPR	FEN		IPR	FEN		IPR	FEN
1	5.0	5.0		100.21	100.49		98.50	99.74		98.32	98.24
2	5.0	10.0		102.00	101.40		99.81	99.45		98.81	101.91
3*	5.0	12.5		98.97	100.42		99.16	98.15		101.09	100.09
4*	6.0	15.0		99.76	101.05		99.75	98.64		100.49	101.49
5*	8.0	20.0		99.20	99.92		98.34	99.02		101.07	99.93
6	10.0	5.0		101.44	100.49		101.25	99.59		101.18	100.82
	Mean ± RSD%			100.27 ± 1.127	100.63 ± 0.475		99.47 ± 0.970	99.10 ± 0.519		100.16 ± 1.160	100.41 ± 1.109

*Different laboratory prepared mixtures prepared at the ratio of dosage forms.

Table S1 statistically compares the developed spectrophotometric methods with the results obtained using the corresponding official methods of IPR and FEN in their pure forms^24^, to assure results validity. The calculated Student t-test and F-test values were found to be less than the tabulated theoretical values. The comparison disclosed that absence of any significant difference between the proposed and official methods.

### Application to real sample analysis of marketed co-formulated metered dose inhaler

No noticeable spectral confusion was observed when measuring IPR and FEN simultaneously in their formulated metered dose inhaler from possible excipients; the results were within the limit (Table 3). This demonstrated that the strategies for mathematical filtration of the target analyte that have been proposed work well and do not require any prior mechanical separation. Additionally, Table 3 shows that the validity of the suggested methods and the effectiveness of the drug extraction were confirmed by positive results from the standard addition technique.

**Table 3:** Quantitative estimation of Ipratropium and Fenoterol in Atrovent® comp HFA inhaler and application of standard addition technique.

Drug	Atrovent ® comp HFA Inhaler (BN. 104,604)		Standard addition technique						
	Found%^a^ ± RSD%		Taken (µg/mL)		Added (µg/mL)		Recovery%^b^		
	ICS method	IDW method	IAM method				ICS method	IDW method	IAM method
IPR	100.57 ± 0.738	100.456 ± 0.760	100.02 ± 1.400	5		2.5	100.76	99.46	101.76
						5.0	101.72	100.76	98.77
						10.0	100.35	98.48	100.66
				Mean ± RSD%			100.94 ± 0.575	99.57 ± 0.936	100.40 ± 1.236
FEN	100.01 ± 0.361	101.58 ± 0.219	99.98 ± 0.396	12.5		6.25	100.76	100.05	99.45
						12.5	100.37	98.40	101.61
						25.0	99.98	100.36	101.81
				Mean ± RSD%			100.37 ± 0.318	99.60 ± 0.862	100.96 ± 1.071

^a^Average of five determinations.

^b^Average of three determinations.

### Application on delivered dose uniformity testing

The unique characteristics of the proposed spectrophotometric approaches, such as being simple and environmentally friendly processes, crucial solvent and energy savings, in addition to the measurement of low IPR and FEN concentrations, increased their suitability for tracking the uniformity of delivered dosage units. Following international guidelines, delivered dose uniformity protocol was applied as described^25,26^. The following formula was used to determine the dosage form acceptance value (AV)^49^

$${\text{AV }} = \, \left| {{\text{M }} - {\overline{\text{X}}}} \right| \, + {\text{ ks}}$$

where M is the reference value, (X̄) is the mean recovery percent for the assayed ten dosage units (two actuations each), k is the acceptability constant which equals (2.4), and S is the standard deviation. Ten units were examined, and the average recovery percentage for each fell between 98.50% and 101.50%. In this case, the reference value (M) equaled the mean recovery percent, so the final acceptance value (AV) was calculated by multiplying the standard deviation of ten analyses by the acceptability constant (k) of 2.4. Table 4 shows that, for all three proposed approaches, the AV values were below the maximum permitted acceptance value (L1), which is less than 15, indicating satisfactory delivered dosage uniformity.

**Table 4:** Results of delivered dose uniformity testing for determining Ipratropium and Fenoterol in Atrovent® comp HFA inhaler using the proposed spectrophotometric methods.

Atrovent® comp HFA meter dose no	Label claim (%)					
	IPR			FEN		
	ICS method	IDW method	IAM method	ICS method	IDW method	IAM method
1	99.99	99.89	100.54	96.88	97.47	100.09
2	103.63	100.26	100.32	98.23	98.44	100.02
3	100.04	98.89	100.97	97.75	102.18	102.23
4	98.37	101.25	100.63	97.26	98.81	100.13
5	102.73	99.52	100.45	98.71	99.03	101.12
6	99.46	103.97	101.44	97.02	103.06	102.31
7	101.32	101.99	95.58	100.16	98.66	95.87
8	103.05	102.26	96.61	99.92	99.42	97.85
9	99.59	103.38	99.94	100.40	100.61	98.53
10	103.05	102.98	102.29	99.92	101.95	101.67
Mean	101.12	101.44	99.90	98.62	99.97	99.98
SD	1.78	1.66	2.0	1.31	1.78	1.95
RSD%	1.76	1.64	2.0	1.33	1.78	1.95
AV *	4.26	3.98	4.87	3.15	4.26	4.69

*Acceptance value = 2.4 × SD with maximum allowed level (L1) is 15.

### Greenness profile, blueness and whiteness evaluation

#### Green solvents selecting tool (GSST)

**Fig. 6:**
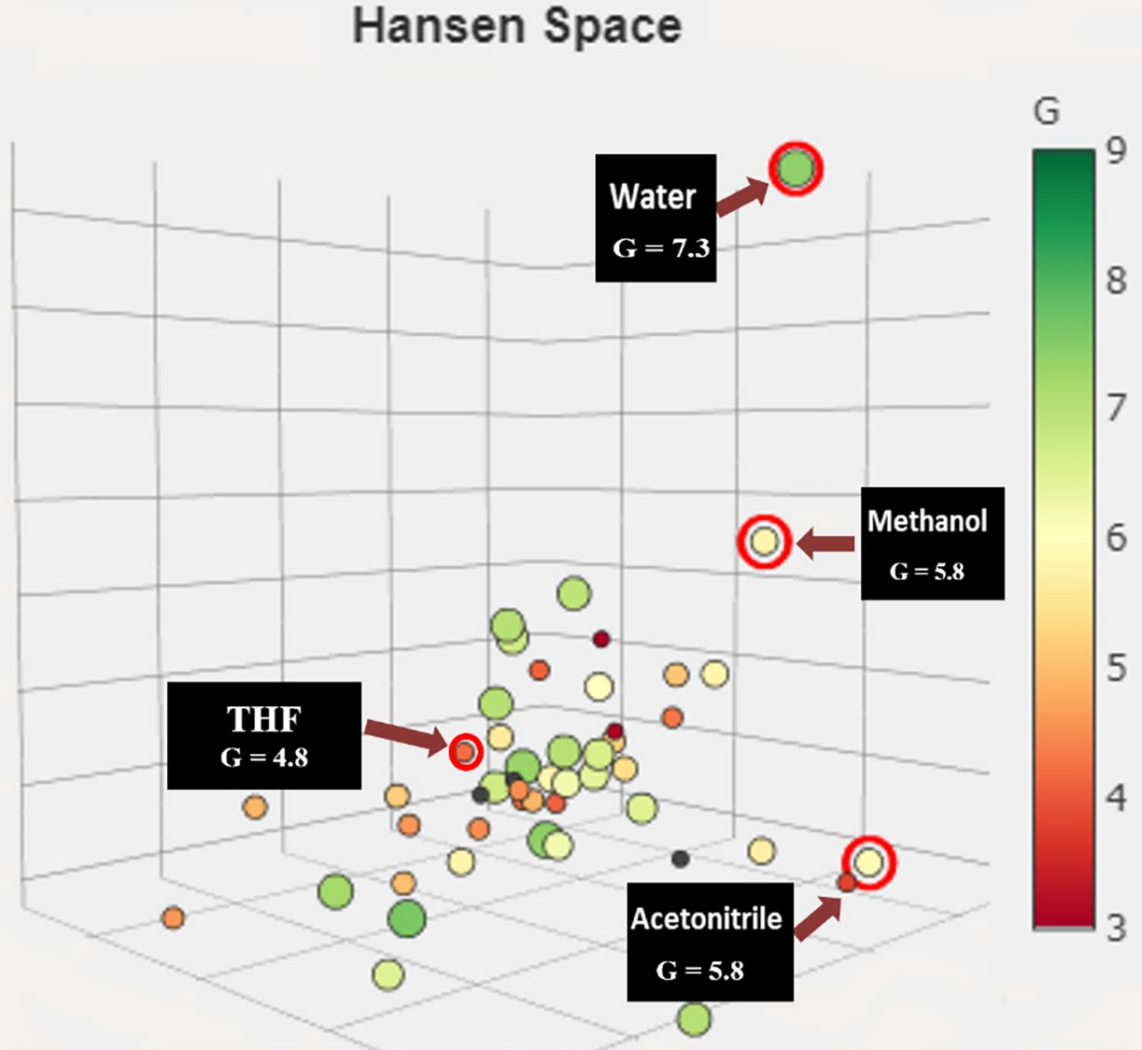
Visualization of the composite score (G) of various solvents using the GSST approach in the Hansen space, represented by spheres of varying sizes colored like traffic lights.

One of the critical factors determining the effect of analytical methods on safety, health, and the environment is the choice of solvent. The main factor in selecting one analytical method over another is a comparison of the solvents used in each. In light of this, Larsen et al*.* recently developed GSST^50^ to offer a simple online framework for comparing different solvents used in printed electronics. Additionally, it helps to make it simple for the users to switch out harmful solvents for sustainable ones by pointing out the available greener alternatives. The GlaxoSmithKline (GSK) Solvent Sustainability Guideline functions as the core for the assessment of GSST. Each solvent is evaluated using a fusion of the four fundamental categories: safety (S), environment (E), health (H), and waste disposal (W). At the final, this is represented diagrammatically by spheres with varying sizes and traffic light colors, marked by a combined score ($G=\sqrt[4]{S\times E\times H\times W}$ ) provided for every solvent within the 1–10 range^50^. Higher G scores and greener-colored sphere sizes correspond to greater solvent sustainability. Here we extended the usefulness of this tool to compare the solvents used in our suggested UV methods against that in the previously reported HPLC method for the analysis of IPR and FEN simultaneously. Figure 6 displays that the proposed methods are more sustainable as the solvent used, water, has the largest size and deep green color sphere, it also has the highest G score (G = 7.3) with category scores: (S = 8.9, E = 8.9, H = 9.5, W = 3.7). On the other hand, the solvents used in the reported HPLC are methanol and tetrahydrofuran (THF), with G scores equal to (G = 5.8) with a small yellow sphere and (G = 4.8) with a smaller orange sphere, respectively. For methanol, the category scores are (S = 7.1, E = 8.4, H = 4.9, W = 4.0), while the values for THF are (S = 4.9, E = 5.2, H = 5.9, W = 3.5). Additionally, in order to select the more environmentally friendly solvent for our proposed UV methods, we compared water with other solvents with very low UV cut-offs, such as methanol and acetonitrile (G = 5.8 with category scores: S = 7.7, E = 8.9, H = 5.9, W = 2.8), and discovered that water has a superior sustainability level. Therefore, water was selected as the solvent for the proposed UV methods due to its sustainability, greenness and safety.

#### Complimentary green analytical procedure index (Complex GAPI)

**Fig. 7:**
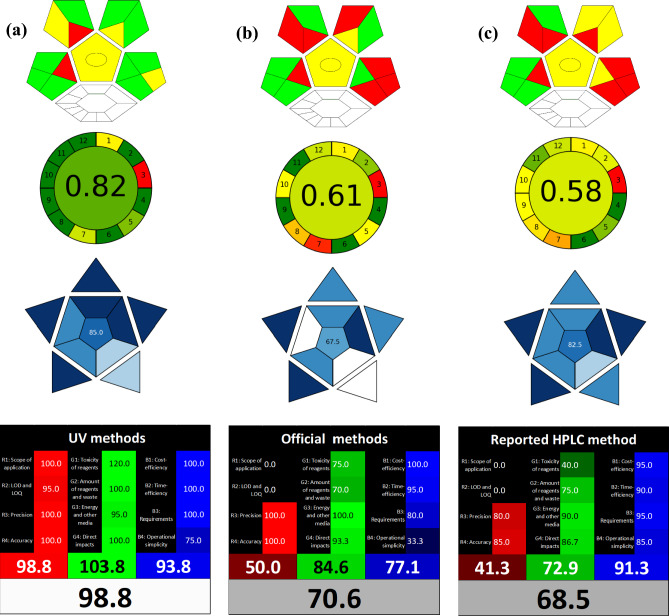
Greenness, blueness and whiteness assessment of (**a**): the proposed spectrophotometric methods, (**b**): official titrimetric methods^24^ and (**c**): reported HPLC method^6^**,** via ComplexGAPI, AGREE, BAGI and white assessment tools.

Three cutting-edge metrics were utilized for evaluating each part of the method of analysis, from conception to finality, since the solvents used are not the only restricted criteria for determining a greenness nature to an analytical method. Complex GAPI, an upgraded version of the popular GAPI tool that was first released by Justyna in 2018^51^, was the initial metric to be applied. To provide an in-depth and semi-quantitative assessment of the entire analytical method, a protocol comprising fifteen parameters was followed. This protocol covered sample preparation, storage, transportation, solvents, and instrumental analysis. A pictogram with a three-color code was applied to quickly and simply visual comparison and evaluation. Afterwards, ComplexGAPI was introduced for dealing with the limitations of any pre-analysis method in context with eleven parameters and to offer an easy-to-use tool in the form of simple computer software^52^. This expansion was shown as a further hexagonal region at the bottom of the GAPI pictogram. When our proposed UV spectrophotometric methods were compared to official potentiometric methods and the published HPLC method, the green color was more prominent in the UV methods, since water has the highest level of greenness in addition to using less energy and waste per sample analysis as shown in Fig. 7. Notably, the additional hexagonal has no color assigned to it because the proposed method not applied pre-analysis processes for sample analysis.

#### Analytical greenness (AGREE) metric

In addition to ComplexGAPI, the recently released AGREE software was used to evaluate methods sustainability in a more quantitative way. The degree to which the method of analysis adhered to each principle of Green Analytical Chemistry (GAC) was assessed and colored, resulting in a circular pictogram with twelve sections that went from deep green to full red. It measures how green various aspects are, including the number of stages, toxicity of the reagents, waste produced, energy requirements, automation, and miniaturization. Ultimately, a score that ranged from zero to one, which is the result of giving each parameter used in the analysis a unique value, was assigned in the center of the circle, where higher values denoted greater sustainability^53,54^. In comparison to the official potentiometric (0.61) and reported HPLC (0.58) methods, the proposed spectrophotometric methods exhibit the highest overall score (0.82), as shown in Fig. 7. Due to the off-line sampling in Sect. 1 (colored yellow) and the off-line nature of their instruments in Sect. 3 (colored red), all methods had the same score in these sections. The proposed UV methods outperformed not only in terms of waste and energy consumption (Sects. 7 & 9), but also in the last three sections due to the use of water as a safe and environmentally friendly solvent during analysis.

#### The blue applicability grade index (BAGI)

Blueness, also known as the blue applicability grade index, is a new and easy-to-use tool. BAGI was created to assess the applicability, strengths, and weaknesses of any analytical method and to determine its practicality^55^. When evaluating the applicability of an analytical method, the BAGI metric tool considers its primary ten attributes. Attributes 1–3 deal with the analytical determination step, attributes 4 and 5 are regarding the sample preparation step, and attributes 6–10 are about both. A pictogram of an asteroid as a graphical representation and a numerical score at the pictogram's center are the two sets of results produced by the BAGI metric tool. The asteroid pictogram uses four different blue sorts to indicate different levels of compliance: dark blue for high, blue for moderate, light blue for low and white for non-compliance. In order to be considered "practical," the method must receive a minimum of 60 points^56,57^. In comparison to the official (67.5) and reported (82.5) methods, the proposed spectrophotometric method received higher score (85). All methods are achievable in practice, as demonstrated by the BAGI pictograms displayed in Fig. 7. The capacity of spectrophotometric techniques to carry out both quantitative and confirmatory analysis, time efficiency and its increased level of automation explain for this higher score.

#### White analytical chemistry (WAC)

Since green methods focus on specific aspects of reducing environmental harm, not all green methods are sustainable in the field of analytical chemistry. More than just being green, sustainability also considers the cost, validity, and effectiveness of the method. As a result, sustainable methods offer an extensive balance between analytical method productivity, performance, and sustainability as well as the ability to identify shortcomings^58^. The RGB12 algorithm tool was used to ensure the sustainability of the methods. It consists of an Excel spreadsheet that can be downloaded for free that allows you to compare up to ten methods at once. The twelve WAC assumptions are covered by three tables: red, green, and blue. The performance of the method analysis is discussed in the red table with respect to application scope, precision, accuracy, and lower limit of detection and quantification. The sustainability of the method is assessed in the green table in terms of waste, energy consumption, and reagent toxicity, while the blue table includes details about cost, time, and operational simplicity. A table featuring three colored columns (red, green, and blue) is used to depict the final summary. The method's white color is created by combining the previously mentioned colors. The table's bottom whiteness score, expressed as a percentage out of 100, represents how closely the WAC postulates are adhered to. The more efficient and sustainable approach would be the higher percentages of the three colors that were then given the highest whiteness score^59^. The enhanced analytical performance of the proposed spectrophotometric methods over the official potentiometric and published HPLC methods for the analysis of pure forms and assay of the marketed dosage form was illustrated by the RGB12 algorithm tool in Fig. 7. Additionally, its ability to test delivered dose uniformity expands its scope of application. Because of their ease of use, low energy consumption, and green credentials, the proposed UV methods were found to be more functional and sustainable.

## Conclusion

Atrovent® comp HFA is a recently developed co-formulated inhaler of IPR and FEN used to treat asthma and COPD. This work represented a successful attempt to develop eco- and user-friendly analytical methods for the simultaneous quantification of IPR and FEN in their new metered dose inhaler. Water-based spectrophotometry was chosen over classical chromatographic ones because it has greater attributes, these include excellent water sustainability, reduced solvent and energy consumption, independence from highly skilled analysts, cost- and time-effectiveness. The multiple inherited spectral challenges of the mixture components, especially IPR, in which conventional spectral resolution strategies were unable to resolve, acted as a barrier to direct spectrophotometric analysis. In order to overcome these challenges, three recently developed and smart spectrophotometric approaches were introduced in this work. This was accomplished by utilizing one or more numerical or spectral factors, such as two absorptivity factors in the ICS method, an equality factor in IDW and absorbance ratio spectrums in IAM, to induce clever mathematical filtration of the desired drug. The methods proposed were effectively applied to ensure the potency of the metered dose inhaler and their delivered dose uniformity. The study also effectively used the GSST metric to evaluate the greenness score of water relative to other widely used solvents. Additionally, the advanced Complex GAPI pictograms and the AGREE tool were used to evaluate the sustainability of the proposed methods, confirming their superiority in terms of sustainability over the official and reported HPLC ones. Lastly, a comprehensive analysis of the proposed methods using the WAC tool was presented, pointing out the highest level of adherence to the WAC establishes.

## Supplementary Information

Supplementary Information.

## Data Availability

All data generated or analysed during this study are included in this published article.

## Abbreviations

AV: Acceptance value
AGREE: Analytical greenness metric approach
BAGI: Blue applicability grade index
Complex GAPI: Complimentary green analytical procedure index
COPD: Chronic obstructive pulmonary disease
DWM: Dual wavelength method
FDA: Food and drug administration
FEN: Fenoterol hydrobromide
FEV_1_: Forced expiratory volume in one second
GAC: Green analytical chemistry
GSST: Green solvents selecting tool
IAM: Induced amplitude modulation
ICH: International council for harmonization
ICS: Induced concentration subtraction
IDW: Induced dual wavelength
IPR: Ipratropium bromide
LOD: Limit of detection
LOQ: Limit of quantification
WAC: White analytical chemistry
