# Supplementary material for: Intelligent spectrophotometric resolution platforms for the challenging spectra of ipratropium and fenoterol in their combination inhaler with ecological friendliness assessment
Source: Sci Rep. 2024 Sep 28;14:22406. doi: 10.1038/s41598-024-72431-x (PMC11436838; doi:10.1038/s41598-024-72431-x)
Supplement: Supplementary file 1 — Supplementary Information. [file 41598_2024_72431_MOESM1_ESM.docx]

Supplementary material

**For**

**Intelligent spectrophotometric resolution platforms for the challenging spectra of ipratropium and fenoterol in their combination inhaler with ecological friendliness assessment**

Salma N. Ali^1^, Samah S. Saad^1^, Ahmed S. Fayed^2^, Hoda M. Marzouk^2,^*

^1^ Pharmaceutical Analytical Chemistry Department, College of Pharmaceutical Sciences and Drug Manufacturing, Misr University for Science & Technology, 6^th^ of October City, Giza, Egypt.

^2^ Pharmaceutical Analytical Chemistry Department, Faculty of Pharmacy, Cairo University, Kasr Al-Aini Street, 11562, Cairo, Egypt

**Theoretical background**

1. ***Induced concentration subtraction method (ICS)***

Recently, the intelligent spectrophotometric method known as the ICS method was developed to analyze two components simultaneously. One of the components expresses spectra extension over a specific wavelength range, and the other has overlapping spectra **[12,41,42].** What makes this approach unique is that it only requires one regression equation to estimate both components in a mixture and deals with the native D^◦^ spectra without the need for additional spectral manipulation steps. Its basic idea is to manipulate two calculating numerical values known as "Absorptivity Factors" in order to perform a mathematical filtration of the desired analyte from its matrix.

Consider a lab mixture of X and Y, where Y has a peak at a specific wavelength (λ_max_) and is spectrally extended than X at that wavelength (λ_ext_). A significant absorbance reading of component X at the selected λ_max_ of Y is a necessary prerequisite.
To use this method, three steps must be completed first:

Step (1): A unified regression equation between absorbance values at λ_max_ versus different concentrations of pure Y is constructed by utilising the D^◦^ absorption spectra of pure Y.

Step (2): The first absorptivity factor (F_1_), which is the average ratios between absorbance values at λ_max_ and λ_ext_ for different concentrations of component Y in its pure form, is only calculated for pure drug Y.

$$F_{1}=\frac{{Absorbance of Y at \lambda}_{max}}{{Absorbance of Y at \lambda}_{ext}}$$

By multiplying F_1_ by the mixture's absorbance value at λ_ext_, which only represents the absorbance value of component Y, one can estimate the absorbance contribution of component Y in any mixture at λ_max_.

Step(3): To calculate the second absorptivity factor (F_2_), divide the absorbance value of component X at the selected λ_max_ by the absorbance value of component Y at the same concentration. As a result, at λ_max_, F_2_ makes it easier to convert mathematically between the absorptivity values of the two components.

$$F_{2}=\frac{{Absorbance of X at \lambda}_{max}}{{Absorbance of Y at \lambda}_{max}} of same conc= \frac{a_{{X (\lambda}_{max})}{.b.c}_{X}}{a_{{Y (\lambda}_{max})}{.b.c}_{Y}}=\frac{a_{{X (\lambda}_{max})}}{a_{{Y (\lambda}_{max})}}$$

At this point, it would be simple to quantify each element in the scanned mixture as follows:

The mixture's absorbance value at λ_ext_, which solely indicates component Y and excludes component X, is measured. To determine the absorbance contribution of Y in the mixture, multiply this value by the F_1_ calculated in step (2). The concentration of component Y in the mixture could be readily estimated upon substitution in the unified regression equation.

To obtain the concentration of the second component (X) in the mixture, the mixture's absorption value at λ_max_ is noted and then substituted into the unified regression equation to obtain the mixture's estimated total concentration. This concentration is then subtracted by obtained concentration of Y in mixture, the net effect of the previous steps is the concentration of component X in the mixture, but it takes the form of component Y's absorptivity constant (before correction). This is explained by the fact that the total concentration of the mixture was estimated by using the unified regression equation that was calculated for the Pure Y component.

Estimated Conc_X_ before correction = Total estimated Conc_mix_ – Conc_Y_

Estimated Conc_X_ before correction = $\frac{{Absorbance}_{X}}{a_{{Y (\lambda}_{max})}.b}$

The estimated concentration value of component X is then multiplied by $( \frac{1}{F_{2}} )$in order to return it to its normal concentration in the mixture.

Final Conc _X_ $= \frac{{Absorbance}_{X}}{a_{{Y (\lambda}_{max})}.b} \times\frac{a_{{Y (\lambda}_{max})}}{a_{{X (\lambda}_{max})}} = \frac{{Absorbance}_{X}}{a_{{X (\lambda}_{max})}.b}$

***2. Induced dual wavelength method (IDW)***

This technique can be used for a binary mixture of X and Y with fully overlapped zero order absorption spectra at two wavelengths, $\lambda1$ and $\lambda2$, in which the conventional dual wavelength method **[45]** is not applicable because the absorbance of the interfering substance between those two wavelengths is not equal (the absorbance difference does not equal zero) **[43]**. The following equations provide an illustration of this:

A_1_$=$A_X1_ $+$A_Y1_ at $\lambda$ 1 (1)

A_2_$=$ A_X2_ $+$ A_Y2_ at $\lambda$ 1 (2)

where A_1_ refers to the mixture's absorbance at λ_1_ (λ_max_ of X), and A_2_ describes its absorbance at any other wavelengths (λ_2_). In order to cancel out the impact of Y at the two designated wavelengths, the equality factor (F_Y_) of pure Y at these wavelengths is determined:

$F_{Y}=\frac{A_{Y1}}{A_{Y2}}$ $\therefore$ $A_{Y1} = F_{Y}A_{Y2}$

By substituting in Eq. (1)

A_1_$=$A_X1_ $+ F_{Y}A_{Y2}$ (3)

By multiply Eq. (2) by F_Y_

F_Y_A_2_$=$ F_Y_A_X2_ $+$ F_Y_A_Y2_ (4)

And (F_Y_A_Y2_) will be cancelled by computing the difference, Eqs. (3) and (4):

$\Delta A$(A_1_$-$ F_Y_A_2_) $=$ A_X1_ $-$ F_Y_A_X2_  (5)

According to Eq. (5), the mixture's absorbance difference depends only on the values of C_X_ and is not affected by the mixture's C_Y_ value.

($\Delta A=$A_1_$-$ F_Y_A_2_) $=$ slope . C_X_ $\pm$ intercept (6)

Regression Equation (6) is used to determine the concentration of X. It is obtained by graphing the absorbance difference values of the pure X zero order spectra at the two selected wavelengths ($\Delta A=$A_1_$-$ F_Y_A_2_) against the corresponding X concentrations.

The same method that can be used to determine the concentration of Y using the pure X equality factor at the two selected wavelengths (F_X_) is used to calculate the concentration of Y.

***3. Induced amplitude modulation method (IAM)***

In order to determine the concentrations of X and Y in their mixtures with severely or partially overlapping spectra, a newly stablished approach is presented **[43,46]**. In these cases, X and Y either show a low absorptivity of isoabsorptive point or lack one, which leads to an error in the concentration calculations using this point. The present method begins by calculating a regression equation that shows the linear relationship between the difference in ratio amplitudes of various concentrations of pure Y at two chosen wavelengths - one of which is λ_max_ (P_max_), the wavelength with the highest amplitude value in the ratio spectrum, and the other is λ_2_ (P_2_) - where both spectra X and Y show overlap; using a normalized concentration of X’ as a divisor, versus the corresponding ratio amplitude of Y at λ_max_, therefore:

$\Delta P=$ slope . P_max_ $+$ intercept (1)

where $\Delta P$ is the difference of ratio amplitudes between λ_max_ and λ_2_, and P_max_ is the corresponding ratio amplitude at λ_max_ of the ratio spectrum.

It is possible to determine P_max_ of pure Y from Eq. (1) by determining $\Delta P$ for the mixture.

The value of P_max_ of X (constant) is obtained by subtracting the P_max_ of Y from the P_max_ of the mixture_(X+Y)_. This constant $( \frac{a_{X}C_{X}}{a_{X}} )$ parallel to the X axis corresponds to the recorded concentration of X.

P_(X)_ $=$ P_(X+Y)_ $-$ P_(Y)_

P_(X)_ $=$ [$( \frac{a_{X}C_{X}}{a_{X}} )$ + $( \frac{a_{Y}C_{Y}}{a_{X}} )$] $-$ $( \frac{a_{Y}C_{Y}}{a_{X}} )$ (2)

$\therefore$ P_(X)_ $=$ C_X_  (3)

The constant value (C_X_) is subtracted from the mixture's ratio spectrum to yield the component Y ratio spectrum.

P_(X+Y)_ $-$ P_(X)_ $=$ P_(Y)_

[C_X_ + $( \frac{a_{Y}C_{Y}}{a_{X}} )$] $-$ C_X_ $=$ P_(Y)_ (4)

$\therefore$ P_(Y)_ $=$ $( \frac{a_{Y}C_{Y}}{a_{X}} )$ (5)

This obtained amplitude of ratio spectrum is modulated by multiplying the obtained amplitude P_(Y)_ from Eq. (5) by the "Absorbance ratio spectrum" which was calculated by dividing the entire X normalized spectrum by the Y normalized spectrum, respectively, $( \frac{a_{X}}{a_{Y}} )$.

P_(Y)_ $=$ $[( \frac{a_{Y}C_{Y}}{a_{X}} )$ $\times$ $( \frac{a_{X}}{a_{Y}} )$] (6)

$\therefore$ P(_Y_) $=$ C_Y_ (7)

The recorded concentration of Y [C_Y_] is represented by the obtained amplitude, which is modulated into a straight line parallel to the X axis.

**Table S1.** Statistical comparison of the results obtained by the proposed spectrophotometric method and official methods for the analysis of Ipratropium and Fenoterol in their pure forms.

| Parameter | IPR | | | |  | FEN | | | |
| --- | --- | --- | --- | --- | --- | --- | --- | --- | --- |
|  | ICS method | IDW method | IAM method | Official method^a^ |  | ICS method | IDW method | IAM method | Official method^b^ |
| Mean | 100.67 | 100.27 | 100.04 | 99.40 |  | 100.67 | 98.91 | 100.69 | 99.40 |
| SD | 1.297 | 1.178 | 0.609 | 0.926 |  | 1.297 | 0.816 | 0.996 | 1.062 |
| n | 6 | 6 | 6 | 6 |  | 6 | 6 | 6 | 6 |
| Variance | 1.682 | 1.388 | 0.371 | 0.857 |  | 1.682 | 0.665 | 0.992 | 1.128 |
| Student's t-test (2.228)^c^ | 1.952 | 1.422 | 1.414 | - |  | 1.856 | 0.896 | 2.170 | - |
| F value (5.05)^c^ | 1.963 | 1.619 | 2.309 | - |  | 1.491 | 1.696 | 1.137 | - |

^a^ IPR is determined by potentiometric titration method by dissolving 0.350 g in 50.0 mL of water and add 3.0 mL of dilute nitric acid and using 0.01 M silver nitrate as titrant with potentiometric detection of end point as per the British Pharmacopoeia **[24]**.

^b^ FEN is determined by titrimetric method by dissolving 0.600 g in 50.0 mL of water and add 5.0 mL of dilute nitric acid and 25.0 mL of 0.1 M silver nitrate, shake and titrate with 0.1 M ammonium thiocyanate using 2.0 mL of ferric alum as indicator until an orange color is obtained as per the British Pharmacopoeia **[24]**.

^c^ The values in parentheses represent the corresponding tabulated values of t and F at p=0.05.
